# MicroRNA-145: a potent tumour suppressor that regulates multiple cellular pathways

**DOI:** 10.1111/jcmm.12358

**Published:** 2014-08-15

**Authors:** Shi-Yun Cui, Rui Wang, Long-Bang Chen

**Affiliations:** 1 Department of Medical Oncology, Jinling Hospital, School of Medicine, Nanjing UniversityNanjing, Jiangsu, China

**Keywords:** microRNA-145, proliferation, invasion, differentiation, angiogenesis

## Abstract

MicroRNAs are endogenous, small (18–25 nucleotides) non-coding RNAs, which regulate genes expression by directly binding to the 3′-untranslated regions of the target messenger RNAs. Emerging evidence shows that alteration of microRNAs is involved in cancer development. MicroRNA-145 is commonly down-regulated in many types of cancer, regulating various cellular processes, such as the cell cycle, proliferation, apoptosis and invasion, by targeting multiple oncogenes. This review aims to summarize the recent published literature on the role of microRNA-145 in regulating tumourigenesis and progression, and explore its potential for cancer diagnosis, prognosis and treatment.

**Table:** 

● Introduction
● miR-145 biogenesis
● Down-regulation of miR-145
● The functions and pathways involving miR-145 targets
– MiR-145 in tumour growth inhibition
– MiR-145 in cancer invasion and metastasis
– MiR-145 in the differentiation of cancer stem cells
– MiR-145 in angiogenesis
– MiR-145 and cancer-associated virus
● MiR-145 in cancer diagnosis and prognosis
● MiR-145 in cancer therapy
● Conclusions and future directions

## Introduction

For a long time, protein-coding genes have been considered the principal effectors and regulators of tumourigenesis. However, this concept has been revised recently. Non-coding RNAs have emerged as active modulators of the protein-coding gene function. Identification of non-coding RNA-based epigenetic regulatory networks has opened up an entirely new area of cancer research.

Among these, microRNAs (miRNAs) are the most-studied non-coding RNAs. They are short, 18–25 nucleotide, non-coding RNAs, which bind to target messenger RNAs (mRNAs), usually in their 3′-untranslated regions (UTR), and inhibit their expression by either inducing their degradation or repressing their translation. Since their discovery in 1993 1,2, over 2500 miRNAs have been identified in the human genome, which are thought to regulate more than 30% of the protein-coding genes. Thus, they have emerged as integral components of nearly every biological process, including cell proliferation, migration, differentiation, apoptosis and angiogenesis 3. Recent evidence has shown that altered expressions of miRNAs are associated with carcinogenesis and development of various cancers 4. By regulating hundreds to thousands of potential target genes, miRNAs form a novel layer of the complicated regulatory network in cells. Thus, disorder of miRNA expression can lead to pathological changes in cells, ultimately contributing to the development of cancers.

MiR-145 was first predicted based on its homology to a verified miRNA from mouse 5, and was subsequently verified in humans, with significantly reduced levels in colorectal cancer 6. Slightly over half (52.5%) of miRNA genes are located in cancer-associated regions or fragile sites in the genome 7. Consistently, miR-145 is located on chromosome 5 (5q32-33), a well-known fragile site in the human genome 8, and is suggested to be co-transcribed with miR-143 9. Down-regulation of miR-145 has been observed in many types of cancers, suggesting that it may serve as a tumour suppressor. So far, miR-145 has been shown to be involved in regulating various cellular processes, such as the cell cycle, proliferation, apoptosis and invasion, by targeting multiple oncogenes. Moreover, reduced expression of miR-145 is associated with a worse prognosis for many cancers, indicating that it may serve as a potential cancer biomarker and an attractive target for cancer therapy. This review aims to summarize recent research on the physiology and pathological functions of miR-145 and its implications for clinical therapy.

## MiR-145 Biogenesis

A unique biogenesis pathway produces miRNAs. RNA pol II transcribes most miRNAs from introns of protein-coding genes as long primary transcripts (pri-miRNAs) with a 5′ m^7^G cap and a 3′ poly-A tail. After transcription, pri-miRNAs undergo at least three steps before becoming the mature single-stranded form.

**Figure 1:**
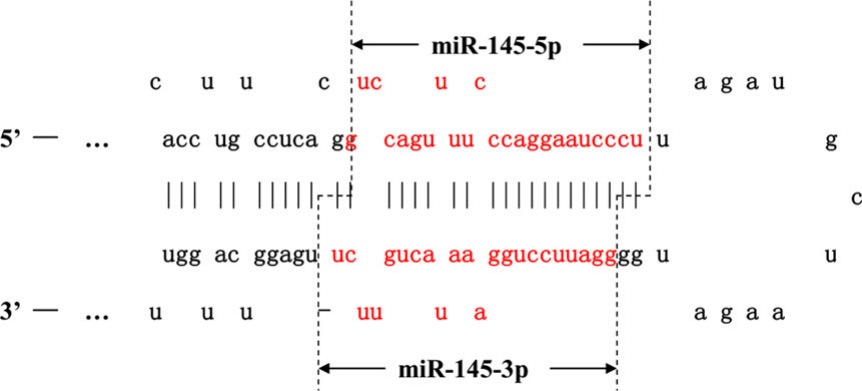
The structure of pre-miR-145. Drosha cuts Pri-miR-145 the stem-loop structural pre-miR-145, and then Dicer removes the loop region from pre-miRNA, leaving the mature sequence. Mature miR-145 has two different forms, namely 5p and 3p, according to which side of the strand they are derived from.

The pri-miRNA is cleaved into a stem-loop structure containing 60–70-nt (precursor) pre-miRNAs by the nuclear microprocessor complex, which comprises the RNase III enzyme Drosha (RNASEN), the DGCR8 (DiGeorge critical region 8), a double-stranded RNA binding protein and multiple RNA-associated proteins such as the DEAD-box RNA helicases p68 and p72 10. P68 and p72 are required for the maturation of miR-145, but not for all miRNAs 11. They enhance pre-miRNA processing by unwinding double-stranded stem loop-containing pre-miRNAs. After nuclear processing, the pre-miRNA is exported into the cytoplasm by RAN-GTPase/Exportin5 (Expo5) for further processing 12. Another RNase type III protein, Dicer, together with its partner TAR (HIV) RNA binding protein (TRBP), removes the loop region from the pre-miRNA and cleaves it into a ∼22-nt double-stranded RNA product containing two forms of mature miRNAs that can be cleaved from either the 5′ or 3′ arm extending out from the stem loop of the pre-miRNA 13–16. For example, mature miR-145 has two different forms, 5p and 3p, according to which side of the strand they were derived from (Fig.1).

The guide strand or mature miRNA is bound by Argonaute (Ago) to form a miRNA-induced silencing complex (miRISC), which targets the 3′-UTR of the complementary mRNAs for regulation, whereas the passenger strand is usually degraded 17. The specificity of miRNA targeting is defined by how complementary are the ‘seed’ sequence (positions 2 to 8 from the 5′end of the miRNA) and the ‘seed-match’ sequence (generally in the 3′ UTR of the target mRNA). A perfect match between miRNAs and mRNA sequences promotes the degradation of mRNAs by Ago1/2, while an imperfect match usually leads to translational repression 18. The imperfect nature of the miRNA:mRNA interaction means that a single miRNA can potentially target tens to hundreds of mRNAs.

## Down-regulation of miR-145 in cancer

Reduced expression of miR-145 has been reported in many types of cancer. The first report, from Michael *et al*., observed low expression of miR-145 in colonic adenocarcinoma compared with mucosa 6. This result was confirmed by further research in different cancer cell lines, including both solid and blood malignancies (Table1). This evidence indicated widespread deregulation of miR-145 levels in various cancers.

**Table 1:** Down-expression of miR-145 in cancers

Cancer types	Technique for detection	References
Lung	Microarray, qRT-PCR, *in situ* hybridization	19
	Microarray, qRT-PCR	20
Breast	Microarray, qRT-PCR	21,22
	Microarray, northern blot	23
	Microarray, qRT-PCR, *in situ* hybridization	24
Ovary	Microarray, northern blot	25,26
	Microarray, qRT-PCR	27
Prostate	Microarray, qRT-PCR	28–31
Colon	Microarray, qRT-PCR	6,32–37
Liver	Microarray	38
	Microarray, qRT-PCR	39–41
	qRT-PCR	42
Biliary tract	qRT-PCR	43
Larynx	Microarray, qRT-PCR	44
Oesophagus	Microarray, qRT-PCR	45–48
Pancreas	qRT-PCR	49
Oral	Microarray, qRT-PCR	50,51
Bladder	Microarray, qRT-PCR	52–55
	qRT-PCR	56,57
Nasopharynx	Microarray, qRT-PCR	58
Glioma	Microarray, qRT-PCR	59
Cutaneous squamous cell carcinoma	Microarray, qRT-PCR	60
Basal cell carcinoma	Microarray, qRT-PCR	61
Liposarcoma	Microarray, qRT-PCR	62
B-cell malignancies	qRT-PCR	63

**Figure 2:**
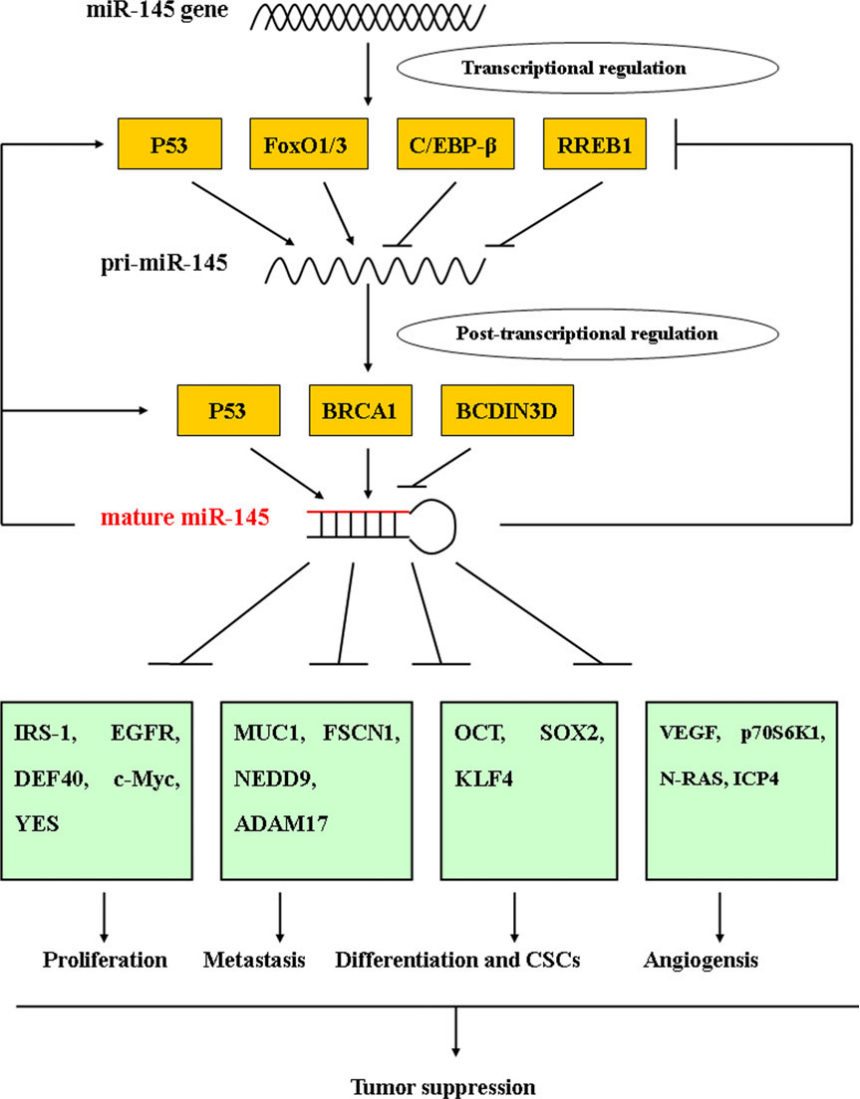
The upstream regulation and downstream targets of miR-145. P53 and FoxO1/3 promote pri-miR-145 transcription, while RREB1 and C/EBP-β inhibit its transcription. P53, BRCA1, BCDIN3D and DDX6 can regulate miR-145 processing at the post-transcriptional level. The downstream target genes of miR-145 include IRS-1, EGFR, c-Myc, MUC1, FSCN1, OCT4 and SOX2. By modulating multiple oncogenes, miR-145 regulates different cellular processes, including proliferation, apoptosis, differentiation, invasion and angiogenesis.

However, the mechanisms responsible for its down-regulation are largely unknown. Gene expression regulation is a multilayered network, where transcription is a major regulatory step for the biosynthesis of miRNAs. A couple of transcriptional factors have been identified that modulate the expression of miR-145, and an epigenetic mechanism also plays an important role in the silencing of miR-145. After transcription, pri-miR-145 undergoes several processing steps before its maturation, where some factors also play essential roles at the post-transcriptional level (Fig.2).

p53 is a master tumour suppressor that controls diverse cellular pathways. Recent evidence indicated that some miRNAs are also regulated by p53, such as miR-34, miR-192/215, miR-107 and miR-145 64,65. Sachdeva *et al*. found that p53 could increase miR-145 expression by directly binding to the p53 response elements-2 (p53RE-2) in the miR-145 promoter, which was possibly the mechanism of p53-mediated repression of c-Myc 66. Low miR-145 expression was demonstrated in both laser capture microdissected prostate tissues and 47 cancer cell lines, coupled with p53 mutations 67. Glucocorticoid-induced human papillomavirus oncoprotein E6 (HPV-E6) activation suppressed p53 and miR-145 expression in cervical cancer 68. The tumour-suppressive effect of miR-145 is dependent on p53 activation in some tumours 69,70. Furthermore, Suzuki *et al*. discovered that p53 could facilitate Drosha-mediated pri-miR-145 processing through an association with the DEAD-box RNA helicase p68 (also known as DDX5), a key component of the Drosha complex, indicating post-transcriptional regulation also plays an important role in the modulation of miR-145 expression by p53 71. Interestingly, there seems to be positive feedback regulation between miR-145 and p53, partially by impairing the murine double minute 2 (MDM2)-p53 feedback loop 70,72. This could partially explain why miR-145 is frequently down-regulated in p53-mutated cancers.

The Kras gene mutation is commonly found in many malignancies, and can stimulate a number of downstream effectors, including the mitogen-activated protein kinase (MAPK) and phosphoinositide-3-kinase (PI3K) pathways, ultimately promoting cellular proliferation, survival and motility 73. Kent *et al*. showed that Kras signalling led to repression of the miR-143/145 cluster by activating Ras-responsive element-binding protein 1 (RREB1) in pancreatic cancers. In turn, miR-143 and miR-145 targeted KRAS and RREB1, establishing a feedback circuit of Ras signalling 74. The same results were also obtained in colorectal cancers 75. Consistent with this, there is an inverse correlation between KRAS and the miR-143/145 cluster in colorectal cancer 76.

However, the reason for the low expression of miR-145 in tumours with wild-type p53 is unclear, implying that factor(s) other than p53 are involved in miR-145 regulation. In addition to p53, other transcription factors participate in the regulation of miR-145, including CCAAT/enhancer-binding protein-beta (C/EBP-β), beta-catenin/T cell factor 4 (TCF4) and forkhead transcription factors of the O class 1 and 3 (FoxO1 and FoxO3) 77–79. In addition, DNA methylation in the upstream sequence of miR-145 contributes to the down-regulation of miR-145 in prostate cancer, and, importantly, interferes with the binding of p53 to the p53 response element in the upstream region of miR-145 67.

Post-transcriptional regulation is also critical for miR-145 expression. Apart from p53, Breast cancer 1 (BRCA1) recognizes the root of the stem-loop structure of pri-miR-145, directly associates with Drosha and DDX5 of the Drosha complex, and interacts with Smad3, p53, and DHX9 RNA helicase, promoting miR-145 processing 80. Similarly, the p38 MAPK-MK2 signalling pathway promotes miR-145 biogenesis by facilitating the nuclear localization of DDX5 81. Conversely, BCDIN3D is a methyltransferase that modifies the 5′-monophosphate end of miRNAs, including pre-miR-145, which affects their recognition by Dicer. BCDIN3D depletion reduced the level of pre-miR-145 and increased the level of mature miR-145 in breast cancer cells 82. DEAD-box RNA helicase 6 (DDX6) preferentially increases the instability of the host gene product of miR-145 (NCR143/145) by promoting the assembly of processing bodies (P-bodies), thus negatively regulating miR-145 expression at the post-transcriptional level 83. Doublecortin-like kinase 1 (DCLK1) post-transcriptionally regulates miR-145 in pancreatic cancer; however, the mechanism is unclear 84.

## The functions and pathways involving miR-145 targets

To date, numerous oncogenic genes have been confirmed as targets for miR-145, which cover multiple biological pathways, including proliferation, invasion, differentiation, angiogenesis and cancer-related viruses, supporting the proposed tumour-suppressor role of miR-145 in cancers (Table2). For example, miR-145 could suppress cancer cell proliferation by targeting growth factor-related genes such as IRS-1, IGF-IR or epidermal growth factor receptor (EGFR) 85–87. Moreover, inducing cell apoptosis and cell cycle arrest by inhibiting DFF45, CBFB, CLINT1, PPP3CA or c-Myc also contributes to miR-145-mediated tumour growth suppression 66,88,89. Direct regulation of some oncogenes involved in cancer cell invasion and metastasis, like MUC1, FSCN1, NEDD9 and SOX9, by miR-145 has been identified in many cancers 90–93. In addition, there is evidence that miR-145 has an important role in regulating cell differentiation by targeting core reprogramming factors, including OCT4, SOX2 and KLF4 94,95. Meanwhile, miR-145 could retard angiogenesis process by targeting VEGF, hypoxia-inducible factor 1α (HIF-1α) or N-RAS 96–98. What is more, some cancer-related virus genes, such as infected-cell polypeptide 4 (ICP4), have been validated as targets for miR-145 99. By regulating these target genes, miR-145 exerts a potent tumour-suppressive effect. However, the number of target genes is still growing, indicating a complicated regulatory network for miR-145.

**Table 2:** The functions and involved pathways of miR-145 targets

Target gene	Involved pathway	Cancer types	References
IRS-1, IGF-IR	Proliferation	Colon cancer, hepatocarcinoma	85,87–89,164
EGFR, NUDT1	Proliferation	NSCLC	90
ILK	Proliferation	Bladder cancer	93
DFF45	Apoptosis	Colon cancer	94
CBFB, PPP3CA and CLINT1	Apoptosis	Bladder cancer	95
TNFSF10	Apoptosis	Prostate cancer	97
SOCS7	Apoptosis	Bladder cancer	98
c-Myc	Proliferation, apoptosis, invasion	Colon cancer, NSCLC, ovarian cancer, breast cancer, oesophageal and oral squamous cell carcinoma	66,99,101–104
FLI1, EWS-FLI1, Ets1(ERG),	Proliferation, apoptosis, invasion	Colon cancer, prostate cancer, gastric cancer, Ewing's sarcoma	106–108,110
RTKN	Proliferation	Breast cancer	112
YES, STAT1	Proliferation	Colon cancer	113
HDAC2	Proliferation	Hepatocarcinoma	114
PAI-1	Unknown	Bladder cancer	115
MUC1	Invasion	Breast cancer, ovarian cancer	121–123
FSCN1	Invasion	Bladder cancer, oesophageal squamous cell carcinoma, prostate cancer, breast cancer, melanoma	124–130
JAM-A	Invasion	Breast cancer	129
SWAP70	Invasion	Prostate cancer	131
HEF1/NEDD9	Invasion	Glioblastoma, prostate cancer, renal cell carcinoma	134–136
N-cadherin	EMT, invasion	Gastric cancer	137
ADD3	Invasion	Glioma	138
ADAM17	Proliferation, invasion	Renal cell carcinoma	140,141
SOX9	Proliferation, invasion, CSCs	Glioma, head and neck cancer	138,142
CTGF	Invasion	Glioma	143
Catenin delta-1	Proliferation, cell cycle, invasion	Colon cancer	144
PAK4	Proliferation, invasion	Colon cancer	145
OCT4	Differentiation and CSCs	Human embryonic stem cells, hepatocarcinoma, breast cancer, NSCLC, endometrial adenocarcinoma, skin keratinocyte	41,93,95,153,155–157,167
SOX2	Differentiation and CSCs	Human embryonic stem cells, glioblastoma	153,154
KLF4	Differentiation and CSCs	Human embryonic stem cells	153
VEGF	Angiogensis, invasion	Osteosarcoma	161
p70S6K1	Proliferation, angiogenesis, invasion	Colon cancer, ovarian cancer	123,162
N-RAS	Angiogenesis	Breast cancer, colorectal cancer	163,164
ICP4	Virus	Prostate cancer	166

### MiR-145 in tumour growth inhibition

The tumour-suppressive function of miR-145 is related to its regulation of cell proliferation. The first report describing tumour growth inhibition by miR-145 was from a study in colon cancer cells, where miR-145 suppressed tumour growth by targeting insulin receptor substrate-1 (IRS-1) 85. MiR-145-mediated tumour growth inhibition was also found in cervical cancer and lung adenocarcinomas 19,100. IRS1 is also involved in miR-145′s ability to suppress cell proliferation, anchorage-independent growth and cell motility in gastric cancer and hepatocellular carcinoma 101. Interestingly, the insulin-like growth factor I receptor (IGF-IR) is also a predominant target of miR-145, by which miR-145 suppresses the tumour growth of colorectal cancer and hepatocellular carcinoma, suggesting that the IGR/IRS1 pathway plays an important role in miR-145-mediated anti-proliferative function 86,102.

Furthermore, Cho *et al*. described strong suppression of tumour growth by miR-145 in lung adenocarcinoma patients with an EGFR mutation 20. They further stated that EGFR and nucleoside diphosphate-linked moiety X-type motif 1 (NUDT1) were regulated by miR-145 87. Similarly, forced expression of miR-145 could suppress EGF-induced growth *in vitro* and tumour xenograft growth *in vivo*, suggesting that the EGFR pathway might be involved in the tumour-suppressive effect of miR-145 103. In addition, EGFR-independent activity of the PI3K/Akt or Ras/ERK pathway contributes to EGFR-targeted agents-resistance in non-small cell lung cancer (NSCLC) cell lines. MiR-145 could enhance the cytotoxicity of gefitinib by reducing the activation of Akt rather than ERK 104. Further research indicated that miR-145 indirectly regulates the Akt pathway by directly targeting Integrin-Linked Kinase (ILK), and co-treatment with miR-143 and miR-145 synergistically inhibited cell growth in bladder cancer cells 105.

MiR-145 induces caspase-3-dependent apoptosis in colon cancer by targeting DNA fragmentation factor 45 (DFF45), which is the substrate of caspase-3 and whose cleavage by caspase-3 during apoptosis releases DFF40, which degrades chromosomal DNA into nucleosomal fragments 88. Ostenfeld *et al*. reported that miR-145 induced caspase-dependent and-independent cell death in human urothelial cancer cells by targeting Clathrin Interactor 1 (CLINT1), core-binding factor β subunit (CBFB) and protein phosphatase 3 catalytic subunit α isoform (PPP3CA) 89. However, two other reports also suggested a role of miR-145 in Tumour Necrosis Factor-related Apoptosis-Inducing Ligand (TRAIL)-induced apoptotic cell death 106,107. In bladder cancer, miR-145 induces interferon (IFN)-beta-mediated apoptosis by targeting the suppressor of cytokine signalling 7 (socs7) 108. Moreover, over-expression of miR-145 increased the cleaved-PARP expression and induced apoptosis in breast cancer cells, by repression of oestrogen receptor-α (ER-α) and up-regulation of the TP53 transcriptional targets, such as p53 up-regulated modulator of apoptosis (PUMA) and P21, indicating that miR-145 activates the TP53 pathway and suppresses ER-α 70.

Sachdeva *et al*. revealed that miR-145 causes cell cycle arrest at G0-G1 phase and a decrease in S-phase, partially through silencing the expression of c-Myc 66. Consistent with these results, the miR-145-induced G1/S-phase arrest is mediated by c-Myc in NSCLC, with downstream regulation of CDK4 109. C-Myc, along with the DNA-binding protein Miz-1, binds to the p21(Cip1) promoter and blocks its transcription induction by p53 and other activators, thus preventing p53-mediated apoptosis 110. Therefore, forced expression of miR-145 could restore p53-induced cell cycle arrest, which was likely to be caused, in part, by silencing c-Myc 66. Moreover, the regulation of c-Myc by miR-145 has been associated with proliferation, apoptosis and metastasis in various cancers, including colon cancer, NSCLC, ovarian cancer, breast cancer, oesophageal and oral squamous cell carcinoma 66,109,111–114, highlighting the potent tumour-suppressive effect of the miR-145/c-Myc pathway.

MiR-145 regulates certain oncogenic transcription factors to suppress cell growth. For example, E-26 (ETS) transcriptional factors are well-known proto-oncogenes with mitogenic and transforming activity 115. Some members of the ETS family, such as the Friend leukaemia virus integration 1 (FLI1) and the v-ets avian erythroblastosis virus E26 oncogene homologue (ERG), were identified as targets of miR-145, and are involved in miR-145-mediated apoptosis and repression of cell proliferation in colon and prostate cancer, respectively 116,117. Moreover, miR-145 regulates the expression of Ets1 and its downstream genes, matrix metalloproteinase-1 (MMP-1) and-9, thus inhibiting the invasion, metastasis and angiogenesis of gastric cancer cells 118. Interestingly, EWS-FLI1 is a chromosome translocation-derived chimeric transcription factor that is the major driver of the proliferation of Ewing's sarcoma 119. The EWS-FLI1 gene shares the same 3′-UTR with the FLI1 gene; therefore, its relationship with miR-145 has been explored in Ewing's sarcoma. It transpired that there is feedback regulation between EWS-FLI1 and miR-145 in Ewing's sarcoma growth regulation 120. MicroRNA/mRNA profiling in ovarian cancer suggested a negative correlation between miR-145 and a transcriptional factor, E2F3, but it is not clear whether there is a direct regulation 121.

In addition, there are multiple oncogenic genes that have been identified as targets of miR-145, including RTKN, YES, STAT1, HDAC2 and PAI-1 122–125, contributing to its tumour growth-suppressive effect.

### MiR-145 in cancer invasion and metastasis

MiR-145 was observed to be differentially expressed in node-negative patients or node-positive gastric cancer patients 126. Similarly, effusion samples contain lower miR-145 levels than primary ovarian cancers, suggesting a role of miR-145 in cancer progression 27. MiR-145 is also a biomarker in the transition from localized prostate adenocarcinoma to metastasis 127. Restoration of miR-145 expression could inhibit the migration and invasion ability of cancer cells 40,128. These reports provide a new insight into miR-145-mediated suppression of invasion and metastasis. Mucin 1 (MUC1) is co-expressed with β-catenin at the invasion front of colorectal carcinoma, and is associated with progression of disease and poor prognosis 129. In addition, MUC1 interacts with β-catenin, promoting invasion of breast cancer 130. Therefore, it is considered as an important metastasis gene. MiR-145 suppresses cell invasion and lung metastasis of breast cancer *in vitro* and *in vivo*, and this effect is, in part, attributed to silencing of MUC1 90,131,132. Recent research also described the relationship between miR-145 and some adhesion or cytoskeleton molecules. For example, Fascin homologue 1 (FSCN1), an actin-binding protein, is a candidate target gene of miR-145. MiR-145 participates in the modulation of proliferation and invasion by targeting FSCN1 in various cancers, including bladder cancer, oesophageal squamous cell carcinoma, prostate cancer, melanoma cells and breast cancer cells 91,133–138. Notably, a more cortical actin distribution, reduced actin stress fibres and filopodia formation, and nuclear rotation were observed in miR-145-transfected breast cancer cells by immunofluorescence microscopy. Their effects were partially attributed to the miR-145-dependent regulation of Junctional Adhesion Molecule A (JAM-A) and FSCN1, which contribute to the cytoskeletal rearrangements and cell motility 137. Similar to FSCN1, another actin-binding protein, the SWAP switching B-cell complex 70 kD subunit (SWAP70), was confirmed as a target of miR-145, which is associated with miR-145's modulation of cell migration and invasion in prostate cancer 139. Human enhancer of filamentation 1(HEF1/CAS-L/NEDD9) is a non-catalytic scaffolding protein that interacts with FAK and Src to create binding sites for effector proteins, such as Rac and the Cas-Crk complex. It has been proposed to be involved in cancer invasion and epithelial-mesenchymal transition (EMT) 140,141. Recent research demonstrated that NEDD9 promotes the invasiveness of glioblastoma under the regulation of miR-145, and low expression of miR-145 in glioblastoma was associated with a more aggressive phenotype 92. NEDD9 also mediated the function of miR-145 in regulating EMT, migration and invasion in prostate cancer and renal cell carcinoma 142,143. MiR-145 also targets N-cadherin, a calcium-dependent cell adhesion molecule associated with an increased invasive potential, suppressing invasion and metastasis, rather than inhibiting cell proliferation in gastric cancer 144. Besides direct regulation, the impact of miR-145 on E-cadherin and N-cadherin expression in glioma is partially caused by its regulation of Adducin 3 (ADD3), a cell adhesion-associated molecule 145. Another target gene, Metalloproteinase 17 (ADAM17), is an important member of the ADAM family involved in cancer invasion 146 that plays an important role in the effects of miR-145 on proliferation and migration in renal cancer and glioma cells 147,148. SRY (sex determining region Y) box 9 (SOX9), a positive transcription factor of ADAM17, is also a target of miR-145 and participates in the regulation of tumour-initiating cell and IL-6-mediated paracrine effects in head and neck cancer 93. In addition, miR-145 regulates glioma cell migration by targeting connective tissue growth factor (CTGF), a member of the CCN family, which decreases the expression and phosphorylation of FAK and the expression of secreted protein acidic and rich in cysteine, two important cell migration-relative pathways 149. Moreover, miR-145 directly targets catenin δ-1 and impairs nuclear translocation of β-catenin by disturbing the nuclear import of p21-activated kinase 4 (PAK4), leading to the down-regulation of downstream target genes c-Myc and CyclinD1, which contribute to the attenuation of cell migration and invasion activities in colon cancer 150. PAK4 was also proposed as a direct target of miR-145 151. The above evidence strongly supports the anti-metastatic role of miR-145 in cancer cells.

By contrast, several reports describe the oncogenic effect of miR-145. Although miR-145 and miR-143 are both down-regulated in colorectal cancer samples compared with normal tissues, miR-145 promoted proliferation and morphology in a metastatic colorectal cancer cell line while miR-143 did the contrary. Bioinformatic analysis found that miR-145 targets are differentially expressed between metastatic and non-metastatic isogenic cell line models 152. MiR-145, along with miR-143, was over-expressed in a serial selected invasive glioblastoma cell line, and knocking down both of them demonstrated a synergistic anti-invasive effect 153. Therefore, the role of miR-145 in metastasis may be tissue-specific, and targeting miR-145 in metastatic cancers requires discreet consideration.

### MiR-145 in the differentiation of cancer stem cells

Cancer stem cells (CSCs) are a minority population of cells within a tumour, and are characterized by self-renewal and high tumourigenic capacity. Recent evidence suggests that CSCs might be formed during the process of EMT, and play important roles in development, recurrence, metastasis and drug resistance of cancer 154,155. Embryonic pluripotent stem cells share a similar capacity for self-renewal and transformation into differentiated cells with CSCs, which are maintained by a group of transcription factors in both mice and human, including OCT4, SOX2 and Kruppel-like factor 4 (KLF4) 156–158. Xu *et al*. revealed a direct link between miR-145 and these core reprogramming factors 94. Further research confirmed the reciprocal negative regulation of miR-145 and OCT4 and SOX2 94,159. Given the role of miR-145 in silencing pluripotency during embryonic stem cell differentiation, it is not surprising that miR-145 also inhibits the proliferation of CSCs and induces differentiation, contributing to suppression of tumour growth, migration and EMT 41,95,160–162. Moreover, forced expression of miR-145 suppressed tumour sphere formation and expression of CSC markers and ‘stemness’ factors, including CD133, CD44, Oct4, c-Myc and Klf4 in prostate cancer cells, and inhibited bone invasion and tumourigenesis of prostate cancer *in vivo* 163.

### MiR-145 in angiogenesis

Angiogenesis is a process by which new blood vessels sprout from existing vasculature. It is vital for tumour growth and metastasis, because cancer cells cannot grow beyond 2 mm^3^ without vascular support 164. MiR-145 exhibits tumour-suppressive functions by modulating angiogenesis. VEGF, one of the strongest angiogenic factors, is a direct target of miR-145, regulating the invasion and metastasis of osteosarcoma cells, which implies a role of miR-145 in modulating tumour angiogenesis 96. In addition, miR-145 decreases HIF-1α expression, a major transcriptional regulator of VEGF in response to hypoxia, as well as decreasing VEGF expression by targeting p70S6K1 in colorectal cancer, leading to the inhibition of tumour growth and angiogenesis 97. In addition to VEGF, N-RAS was identified as a target of miR-145, which plays a pivotal role in its anti-angiogenic effect in breast cancer 98. Further research showed that miR-145 inhibits N-RAS and IRS1 expression to suppress AKT and ERK1/2 activation and VEGF expression in colorectal cancer 165.

### MiR-145 and cancer-associated virus

Surprisingly, miR-145 plays an important role in interfering with the cancer-associated viruses-mediated tumourigenesis process. In head and neck squamous cell carcinoma (HNSCC), the HPV-positive (HPV+) samples had a different miRNA profile from the HPV-negative (HPV−) samples, which was more similar to the miRNA profile of cervical squamous cell carcinoma than HPV-HNSCC. Among the HPV core miRNAs was the miR-143/145 cluster, suggesting miR-145 as a potential HPV-specific tumour marker 166. In addition, miR-145 could target a herpes simplex virus-1 essential viral gene, ICP4, to create CMV-ICP4-143T and CMV-ICP4-145T amplicon viruses, leading to restriction of viral replication and oncolysis of prostate cancer cells while sparing normal tissues, both *in vitro* and *in vivo* 99.

## MiR-145 in cancer diagnosis and prognosis

Specific miRNAs have been found to be differentially expressed in the majority of tumour cases; therefore, miRNA expression patterns are capable of distinguishing between malignant and non-malignant tissue. MiR-145 is differentially expressed in breast cancers and normal breast tissues, and its down-regulation is closely associated with invasive breast cancer pathobiological features 23. A noticeable decrease in miR-145 expression was observed in hyperplastic, probable pre-neoplastic, ducts present in normal breast tissues compared with normal ducts, as well as in carcinoma *in situ* and invasive carcinoma, compared with normal tissues 24. In another study, miR-145 was identified as one of the eight basal cell type-specific miRNAs in breast cancer 168. In addition, Wach *et al*. demonstrated that miR-145 was the best discriminating miRNA that could correctly classify 71% of prostate cancer tissue samples and, when combined with miR-375 and miR-143, the correct classification rate of miR-145 reached almost 78%, suggesting that miR-145 could serve as valuable biomarker for the diagnosis of prostate cancer 169. Another independent study obtained an area under the curve (AUC) of 0.74 for the ability of miR-145 expression to discriminate between prostate cancer and non-tumour tissues 30. MiR-145 can also distinguish between subtypes of certain tumours, such as intestinal-type and diffuse-type gastric cancers 170; primary central nervous system lymphomas and nodal diffuse large B-cell lymphomas 171; clear-cell renal cell carcinoma and papillary renal cell carcinoma 172; and different subtypes of liposarcoma 62.

Furthermore, as a non-invasive, blood-based diagnostic tool, cell-free miRNAs have received much interest in recent years. Serum miR-145 has a distinct level in cancer patients compared with healthy ones, suggesting that detection of serum miR-145 has potential as a novel method for early cancer diagnosis 173,174. Moreover, recent evidence has revealed that a combination of circulating miRNAs biomarkers show better sensitivity and specificity for cancer diagnosis. For example, in two independent studies, a combination of plasma markers miR-145 and miR-451, or a combination of miR-145, miR-155 and miR-382, were suggested to increase the sensitivity and specificity for discriminating breast cancer from healthy controls 175,176. Likewise, circulating miR-145 combined with three other circulating miRNAs (miR-20a, miR-21 and miR-221) significantly identified aggressive prostate cancer patients, with an AUC of 0.824 177. Similarly, the combination of three plasma miRNAs (miR-21, miR-145 and miR-155) demonstrated strong potential as a diagnostic marker for early detection of lung cancer, with an AUC of 0.847 178. Moreover, cell-free miRNAs in other body excretions provide a novel approach for cancer diagnosis. The miR-145 level in urine was able to distinguish bladder cancer patients from non-cancer controls (77.8% sensitivity and 61.1% specificity for non-muscle invasive bladder cancer, AUC 0.729; and 84.1% and 61.1% for muscle invasive bladder cancer, respectively, AUC 0.790) and was significantly correlated with grade 179. Li *et al*. also explored the value of faecal miR-145 expression for colorectal cancer diagnosis 180.

On the other hand, many reports have shown that miRNAs, including miR-145, are associated with the clinical outcome of human cancer patients. Time to relapse (TTR) was significantly shorter for NSCLC patients with low miR-145 expression compared with those with high levels. Furthermore, the combination of low miR-145 with p53 mutations was an independent marker of shorter TTR 181. In a study of 527 stage I NSCLC patients, low expression of miR-145 was correlated with brain metastasis 182. Huang *et al*. determined that down-regulation of miR-145 was associated with advanced stage and lymph node metastasis in small cell carcinoma of cervix 183. In addition, miR-145 expression levels in colorectal cancer were associated with tumour stage, depth of invasion (pT category), lymph node status (pN category), development of distant metastases, grade of tumour differentiation, maximal tumour diameter, anatomical localization and serum carcinoembryonic antigen levels, suggesting its potential role as a prognostic marker of colorectal cancer 184. In addition, miR-145 expression is able to predict the response of cancer cells to certain antitumour agents. For example, rectal cancer patients with a low intratumoural post-therapeutic expression of miR-145 had a significantly worse response to neoadjuvant therapy with 5-FU and 50.4 Gy radiation compared with those with high expression, suggesting miR-145 could serve as a response-predicting and prognostic marker in the course of neoadjuvant-treated colorectal cancer 185. Likewise, a profile of five serum miRNAs (miR-20a, miR-130, miR-145, miR-216 and miR-372) was identified as a biomarker to predict the chemosensitivity of colorectal cancer 186. However, in a study of 98 primary colorectal specimens, along with the corresponding normal mucosa specimens, miR-145 down-regulation had no relationship with other clinicopathological features, except the cancer site 187. Results from another group also found no outcome or clinicopathological relevance of miR-145 in colorectal cancer patients 188.

## MiR-145 in cancer therapy

The observation of decreased levels of tumour-suppressive miRNAs in cancers has led to the concept of miRNA replacement therapy. Recently, encouraging results have been achieved in several animal models 189–191. A systemic or local application of a polyethylenimine-mediated delivery of unmodified miR-145 in a mouse model of colon carcinoma obtained a 40% or 60% decrease in tumour growth, respectively, with concomitant repression in ERK5 and c-Myc protein levels compared with negative controls 192. Moreover, delivery of miR-145 mimics by mesenchymal stem cells significantly decreased the migration of glioma cells and the self-renewal of germline stem cells 193.

On the other hand, MiR-145 can influence the sensitivity of tumours to chemo-or radiation therapy, and a combination of miR-145 and chemo-or radiation therapy represents a novel antitumour strategy. Treatment with miR-145 inhibited gastric cancer cell growth and increased its sensitivity to 5-FU 194. Similarly, an adenoviral constructed miR-145 was injected into breast cancer orthotopic mouse models intratumourally, resulting in significant suppression of tumour growth. Furthermore, a treatment combining adenoviral miR-145 and 5-FU produced enhanced retardation of tumour growth, compared with treatment with either drug alone 112. In addition, miR-145 is able to enhance sensitivity to some molecular targeted drugs, including gefitinib and vemurafenib 104,195. Ikemura *et al*. confirmed that MDR1 mRNA was a direct target of miR-145 196. However, further studies are still required to elucidate the mechanisms underlying the miR-145-mediated regulation of resistance to chemo-or radiotherapy.

## Conclusions and future directions

In summary, much evidence indicates that miR-145 represents a potent tumour suppressor, and aberrant miR-145 expression is found commonly in different cancers. Its tumour-suppressive function can be attributed to its regulation of target genes involved in multiple pathways, including proliferation, invasion, angiogenesis and CSCs. MiR-145 is closely associated with many signalling pathways; therefore, the discovery of novel targets is still required to obtain a comprehensive knowledge of the biological roles of miR-145.

The close association between low expression of miR-145 and poor prognosis in cancer patients makes it a valuable prognostic biomarker. Moreover, its potential for cancer diagnosis has also been supported by many studies. More appealing is miR-145-based therapy. MiR-145 treatment can improve the sensitivity to chemotherapy agents in cancer cells, providing a new strategy for overcoming drug resistance. Furthermore, recent progress in drug-delivered systems provides a technical solution for the application of miRNA-based therapy to pre-clinical research. Currently, the application of specific miRNA mimics has demonstrated a great tumour-suppressive capacity in experimental tumour models, such as let-7 or miR-34 189–191. Research into miR-145-based therapy, however, is at an early stage, and effective delivery to the target tissues and drug safety remain challenges. Further investigation of miR-145 may lead to novel therapeutic strategies.
